# Preparation-Dependent Microstructure and Hydrogen Storage in High-Entropy Alloys

**DOI:** 10.3390/molecules31101578

**Published:** 2026-05-09

**Authors:** Chen Chen, Quanhui Hou, Yunxuan Zhou, Zhao Ding

**Affiliations:** 1 Department of Mechanics, Jinzhong University, Jinzhong 030606, China; chenchentgzy@163.com; 2 School of Automotive Engineering, Yancheng Institute of Technology, Yancheng 224051, China; hqhdyx66@ycit.edu.cn; 3 Lanxi Magnesium Materials Research Institute, Lanxi 321100, China; yunxuanzhou@cqu.edu.cn; 4 College of Materials Science and Engineering, National Engineering Research Center for Magnesium Alloys, National Innovation Centre for Industry-Education Integration of Energy Storage Technology, Chongqing University, Chongqing 400044, China

**Keywords:** high-entropy alloys, solid-state hydrogen storage, microstructure, preparation route, refractory alloys, mechanical alloying

## Abstract

High-entropy alloys (HEAs) have emerged as an important class of materials for solid-state hydrogen storage because their compositional complexity provides access to diverse phase constitutions, local lattice environments, and hydrogen-related responses. However, hydrogen-storage behavior in these alloys cannot be understood from composition alone. What ultimately governs performance is the microstructural state generated during preparation. This perspective examines HEAs from that standpoint, focusing on how different preparation routes produce distinct structural states and how those states determine hydrogen accommodation, diffusion, phase transformation, and reversibility. Arc melting and subsequent homogenization typically generate bulk refractory alloys with comparatively simple average phase constitution, whereas mechanical alloying and reactive ball milling produce defect-rich, fine-scale, and metastable non-equilibrium structures. Representative systems are discussed to show that even alloys with similar nominal compositions may follow different hydriding pathways once their structurally realized state changes. The article further evaluates the structural descriptors most often invoked in the field, including phase constitution, local lattice environment, grain size, defect density, interface density, chemical homogeneity, and processing history. It is argued that future progress will depend less on continued composition screening alone than on establishing more transferable microstructure–hydrogen-storage relationships across route-defined structural states.

## 1. Introduction

Solid-state hydrogen storage remains one of the most important routes in hydrogen-energy research because it combines comparatively high volumetric hydrogen density with favorable safety during storage and transport, and therefore continues to be considered alongside compressed and liquefied hydrogen as a practical storage strategy [1,2]. Within this broader context, high-entropy alloys (HEAs) have emerged as a distinctive materials platform because their multicomponent nature opens access to structural states and hydrogen-related responses that are difficult to realize in conventional binary or ternary alloys. Over the past decade, HEAs have been investigated not only as hydrogen-permeable metallic systems, but also as hydride-forming materials and, more broadly, as compositionally complex alloys whose hydrogen-storage behavior can be tuned through phase constitution, local structure, and processing history [3,4]. The rapid expansion of this field has already produced several broad reviews covering composition design, synthesis, characterization, and application prospects from a general hydrogen-storage perspective. The purpose of the present review is narrower. It starts from the observation that hydrogen-storage behavior in HEAs cannot be adequately understood from nominal composition alone, because hydrogen does not interact with an alloy at the level of formula, but with the structural state that the alloy actually presents after preparation. In practice, alloys containing similar constituent elements may still display markedly different activation characteristics, hydrogen capacities, plateau features, sorption kinetics, and reversibility once their preparation route or post-treatment history changes. This is because hydrogen responds not only to elemental identity, but to the phase constitution through which those elements are organized, the local lattice environments they generate, and the defects, interfaces, and metastable structural features retained during processing. This need for a microstructure-centered perspective is especially strong in HEA hydrogen storage because preparation does not merely refine a pre-existing host; it determines which hydrogen-hosting state is actually realized. Depending on synthesis and post-treatment, nominally similar alloys may enter hydrogenation as chemically homogenized bulk phases, as segregation-affected cast structures, as defect-rich nanocrystalline aggregates, or as interface-dominated composites. These route-defined states differ not only in phase constitution, but also in retained strain, local chemical partitioning, defect density, and the continuity of hydrogen-accessible pathways. For that reason, the present article is concerned less with cataloging compositions than with clarifying how preparation-generated structural states become hydrogen-storage behavior.

Seen from this perspective, microstructure is not a secondary detail appended to alloy chemistry. Relative to classical intermetallic or BCC alloy hydrides, the interest of HEAs does not lie in a universally superior storage capacity, but in their ability to combine multicomponent local environments, route-sensitive defect structures, and tunable hydrogenation pathways within a single host framework. In this sense, HEAs are best viewed not as direct replacements for all established hydrides, but as a structurally richer class of materials in which capacity, kinetics, reversibility, and pathway control can be co-tuned through composition and processing together. It is the level at which alloy design becomes hydrogen-storage function. Phase constitution affects the broad type of hydrogen-hosting environment; local lattice environment influences hydrogen occupancy and transport energetics; grain size, defect density, and interface density shape diffusion distance and reaction kinetics; and chemical homogeneity determines whether hydrogen encounters a relatively continuous host lattice or a structurally partitioned landscape with different local affinities and barriers. What remains particularly scarce in the literature is a strict route-controlled comparison in which the same nominal alloy is prepared by two different processing routes and then evaluated on a common hydrogen-storage basis. Such comparisons would be especially valuable because they would separate chemistry from route-generated structure more cleanly than cross-system comparisons. On the basis of the representative studies discussed here, activation behavior and sorption kinetics appear to be the observables most immediately sensitive to microstructural change, whereas maximum reversible capacity depends more strongly on whether the resulting structural state can preserve pathway integrity during cycling. Nonequilibrium states therefore matter for long-term stability not simply because they may accelerate uptake initially, but because they can relax, coarsen, segregate, or partially transform during repeated hydriding/dehydriding cycles, thereby shifting the alloy away from the structural state that first enabled favorable hydrogen storage. Preparation route therefore matters not as a matter of synthetic detail, but because it defines which of these structural states is actually realized in the finished alloy. This review is organized around that idea. Rather than treating HEAs primarily as a large compositional library for hydrogen-storage screening, it examines them as route-generated structural states whose hydrogen-storage behavior is controlled by a preparation-dependent microstructure. The discussion therefore focuses on how different preparation routes generate distinct structural conditions, how those conditions shape hydrogen accommodation, diffusion, phase transformation, and reversibility, and why a more explicit microstructure-centered framework is needed if hydrogen-storage HEAs are to be compared on a genuinely structural basis. Systems in which HEAs act as modifiers in Mg-based materials are considered only as a limited extension of the same logic, rather than as the main identity of the article.

**Figure 1:**
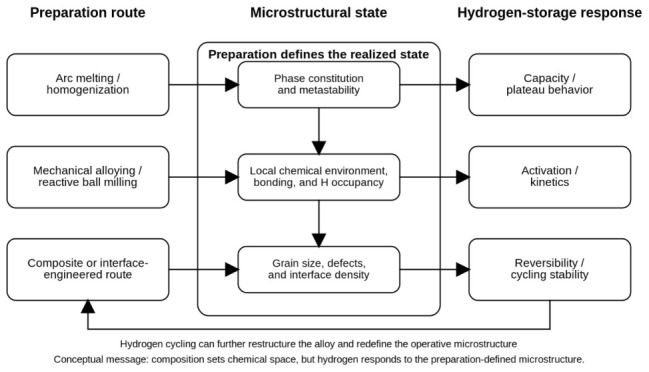
Conceptual framework of a microstructure-centered perspective on hydrogen storage in high-entropy alloys. Preparation route determines the realized microstructural state, including phase constitution, local chemical environment, defect population, and interface density. These structural variables together control hydrogen-storage response in capacity, kinetics, reversibility, and cycling stability. The bottom feedback path emphasizes that hydrogen cycling can in turn restructure the alloy and shift the operative microstructure over time. The often-invoked sluggish-diffusion effect is not singled out here because, for hydrogen storage, diffusion is treated as a microstructure-dependent consequence of the realized route-generated state rather than as a universal HEA-wide attribute.

The scope of this perspective is intentionally selective rather than exhaustive. The literature discussed below is chosen not to catalog all hydrogen-storage HEAs, but to isolate representative cases in which preparation route, structurally realized state, and hydrogen-storage response can be connected in a mechanistically meaningful way. Systems are therefore included when they help clarify one of three issues: how a specific preparation route generates a distinct microstructure, how that microstructure changes the hydrogen-hosting environment, or how contrast among related systems reveals the limits of composition-only interpretation. This boundary is deliberate. To broaden coverage without losing the perspective focus, the revised comparative table also includes additional representative systems from bulk refractory, non-equilibrium, and HEA-modified categories so that the route–microstructure argument is supported across a wider alloy set. The purpose of the present article is not to provide another composition-by-composition survey, but to construct a transferable framework for reading the field through preparation-defined microstructural states. All adapted figures in this perspective are used only to visualize specific route-derived structural states or representative hydrogen-storage responses that are explicitly interpreted in the main text. They do not replace the article’s own synthesis, which is carried primarily by the comparative argument across Section 2, Section 3, Section 4 and Section 5 and by the original conceptual Figure 1. This preparation-dependent logic is summarized schematically in Figure 1. In this schematic framework, no separate sluggish-diffusion block is introduced, because hydrogen transport is interpreted here through the realized microstructure—especially local site landscape, grain scale, interface density, and retained defect structure—rather than through a single generic HEA effect.

## 2. Why Microstructure Matters in Hydrogen-Storage High-Entropy Alloys

The importance of microstructure in hydrogen-storage HEAs begins with a basic point that is often acknowledged but not always carried through in interpretation: hydrogen responds to the structural state obtained after preparation, not to nominal composition in isolation. In principle, HEAs offer an exceptionally broad compositional space and therefore a wide range of possible hydrogen-storage responses. In practice, however, alloys with similar nominal chemistries may still display markedly different activation behavior, hydrogen capacity, sorption kinetics, plateau characteristics, and reversibility once their structural state changes. This is because hydrogen absorption and desorption are governed not only by elemental identity, but also by the phase constitution through which those elements are organized, the local lattice environments they generate, and the defects, interfaces, and metastable features retained during processing [3,4]. Microstructure is therefore the level at which alloy chemistry is translated into an effective hydrogen-hosting environment.

**Figure 2:**
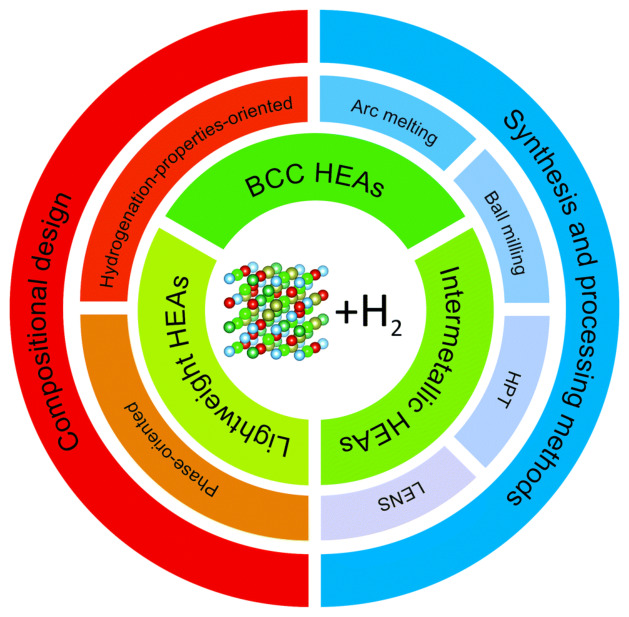
Literature-based overview used here to show that hydrogen-storage high-entropy alloys are realized through distinct route-defined structural classes rather than through a single generic alloy category. Compositional design, synthesis or processing route, and the resulting structural state jointly determine the hydrogen-hosting environment discussed in this perspective. Adapted from ref. [3].

The first reason it matters is that hydrogen-storage HEAs do not constitute a structurally uniform materials class. As summarized in Figure 2, they are realized through different design pathways and preparation histories, and these routes lead to different hydrogen-hosting states rather than to a single generic HEA structure [3]. This distinction is more important than it may first appear. From the standpoint of hydrogen storage, the relevant question is not simply which elements are present, but how those elements are structurally organized when hydrogen first encounters the alloy. A bulk refractory BCC host, a nanocrystalline milling-derived alloy, and a metastable multiphase or partially amorphous system may all be described as high-entropy alloys, yet they do not present equivalent structural conditions for hydrogen accommodation, transport, or release.

Phase labels, however, are only the starting point. Even within the same nominal phase field, the local lattice environment may differ substantially because of atomic-size mismatch, chemical disorder, and nonuniform bonding. This is why descriptors such as atomic-size difference, mixing enthalpy, and valence electron concentration are widely used in HEA research [5,6,7]. Their value here is not that they directly predict hydrogen-storage performance, but that they help rationalize which structural states are likely to form and how stable those states may be once hydrogen is introduced. For hydrogen storage, their deeper significance lies in the kinds of local environments they imply, including interstitial-site geometry, site-energy distribution, lattice stiffness, and the propensity for hydrogen-induced structural change [8]. In other words, such parameters become useful only when they are interpreted structurally rather than treated as stand-alone numerical predictors.

Microstructure matters for another reason as well: hydrogen-storage behavior in HEAs is strongly path dependent. Defects, interfaces, and metastable structural features are not incidental details, but variables that directly affect activation, diffusion, and phase transformation. Point defects and strained regions may create favorable pathways for hydrogen ingress or lower local kinetic barriers, whereas segregation, secondary phases, or overly stable compound-like regions may hinder hydrogen penetration or reduce reversibility. Grain refinement and interface multiplication can shorten diffusion distances and accelerate reaction kinetics, but they may also introduce non-equilibrium states that evolve during cycling. Chemical homogeneity is equally important, because from the standpoint of hydrogen storage the relevant question is not simply whether the alloy is multicomponent, but whether hydrogen encounters a relatively continuous host lattice or a structurally partitioned landscape with different local affinities and barriers.

For this reason, microstructure is more informative than composition alone because it lies closer to the actual controlling hierarchy of hydrogen storage. Composition defines the available chemical space, but microstructure determines how that space is physically realized: which phases form, how atoms are arranged locally, where hydrogen can enter, how far it must diffuse, and what transformations are required for storage and release. This point can be stated more precisely in chemical terms. Hydrogen in a multicomponent alloy does not respond only to average lattice size or average phase identity; it samples a distribution of local interstitial neighborhoods with different electronic surroundings, coordination geometries, and local elastic constraints. In practice, this means that two alloys sharing the same nominal phase label may still differ in how strongly hydrogen is stabilized, how easily it migrates, and how readily hydride formation or decomposition proceeds. For hydrogen storage, the relevant question is therefore not only which average phase is present, but how the local chemical and structural environment around hydrogen is diversified, stabilized, or partitioned by the microstructure created during preparation. The next section therefore turns to preparation routes, not as a matter of synthetic detail, but as the point at which nominal alloy design becomes a specific structural state.

## 3. Preparation Pathways and the Microstructures They Generate

If microstructure matters in hydrogen-storage HEAs, the next question is how it is actually produced and stabilized. Preparation route is not a procedural footnote. It determines whether the alloy enters hydrogenation as a homogenized bulk phase, a defect-rich nanocrystalline aggregate, a metastable solid solution, or an interface-dominated composite. Those route-defined states differ not only in grain size and phase constitution, but also in defect density, local chemical fluctuation, retained strain, and interface population. Because hydrogen responds to this realized structural state rather than to nominal composition alone, changing the route can redirect the same chemistry toward different storage pathways.

### 3.1. Arc Melting and Subsequent Homogenization

**Figure 3:**
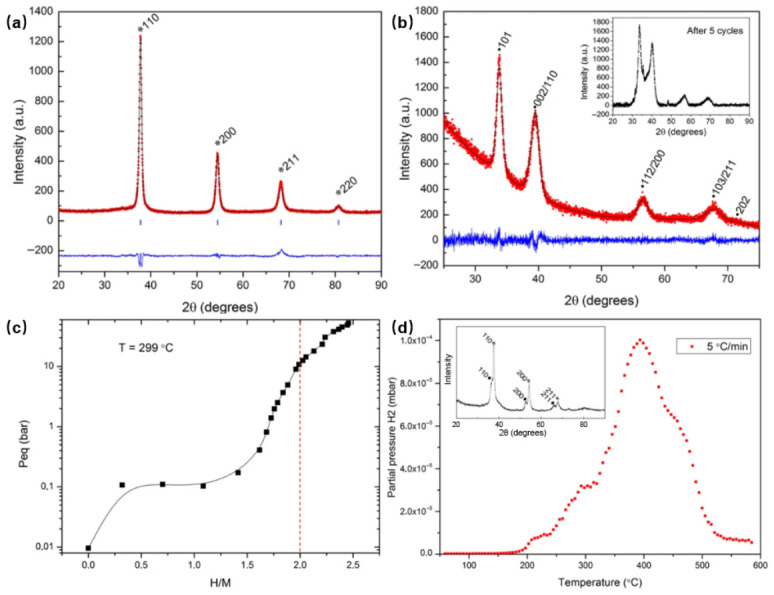
Representative arc-melted refractory HEA used here to illustrate a bulk hydride-forming structural pathway in which hydrogen uptake is coupled to reversible lattice transformation. (**a**) XRD pattern of as-synthesized TiVZrNbHf with a BCC starting structure; (**b**) XRD after hydrogenation, showing formation of a hydrogenated BCT-type phase; (**c**) isothermal hydrogen absorption at 299 °C, giving a maximum capacity of about 2.7 wt.% H_2_; (**d**) thermal desorption and corresponding XRD evidence for recovery of a BCC-type structure after dehydrogenation. Adapted from ref. [9].

Arc melting remains one of the most widely used routes for preparing bulk HEAs for hydrogen storage, particularly for refractory systems based on Ti, Zr, Nb, V, Hf, and Ta. Its appeal lies in the ability to produce chemically complex alloys with relatively simple average phase constitution, most often BCC solid solutions, while retaining sufficient compositional flexibility to tune hydrogen affinity and structural stability. At the same time, the microstructure generated by arc melting is not defined by melting alone. Remelting cycles, homogenization, cooling history, and any subsequent heat treatment strongly affect chemical uniformity, grain structure, and the retention or elimination of local inhomogeneity. Where solidification leaves dendritic chemical segregation insufficiently homogenized, hydrogen diffusion is generally hindered rather than promoted, because the host lattice becomes chemically partitioned into regions with different local affinities, barriers, and transformation tendencies. In this sense, post-melting homogenization is not merely metallurgical cleanup; it is part of establishing a more continuous hydrogen-hosting pathway. The hydrogenation mechanism of arc-melted HEAs should therefore be interpreted as jointly controlled by composition and solidification history. Composition sets the broad thermodynamic window for hydride formation, whereas retained strain, local segregation, and cooling-in defects bias nucleation, interstitial accessibility, and the reversibility of the transformation route. The same logic also explains why classical decrepitation is more likely to be expressed in bulk arc-melted hosts than in pre-comminuted milled powders: in bulk alloys, hydrogen-induced expansion must be accommodated over larger coherent volumes, whereas in milled materials the structural penalty is manifested less as macroscopic fracture and more as local relaxation, coarsening, or loss of non-equilibrium character. Rapid cooling may further leave residual lattice strain, which can transiently lower insertion barriers but can also destabilize hydride cycling if the stored elastic energy is released through fragmentation or irreversible structural redistribution. Arc-melted HEAs should therefore not be regarded as structurally equilibrated by default. Even in bulk alloys that are nominally described as single-phase BCC materials, hydrogen-storage behavior still depends on the extent to which processing has stabilized or preserved a particular structural state [3,4]. This logic can be read directly from the arc-melted TiVZrNbHf system shown in Figure 3, which is used here as a representative bulk hydride-forming example rather than as a stand-alone performance record. The alloy is obtained as a single-phase BCC high-entropy alloy in the as-synthesized state, transforms to a hydrogenated BCT-type structure during hydrogen uptake, and exhibits representative isothermal absorption behavior at 299 °C with a maximum capacity of about 2.7 wt.% H_2_. Equally important, thermal desorption is accompanied by recovery of a BCC-type structure after dehydrogenation. The significance of this example does not lie only in the magnitude of hydrogen uptake. It lies in showing that even for bulk refractory HEAs produced by a comparatively conventional thermal route, hydrogen-storage behavior is inseparable from the structural pathway realized during hydrogenation and dehydrogenation. In this sense, arc-melted HEAs should not be treated as composition-defined materials whose hydrogen response can be inferred from average phase labels alone; they are route-generated structural states whose storage behavior depends on how the host lattice transforms under hydrogen exposure [9].

### 3.2. Mechanical Alloying and Reactive Ball Milling

**Figure 4:**
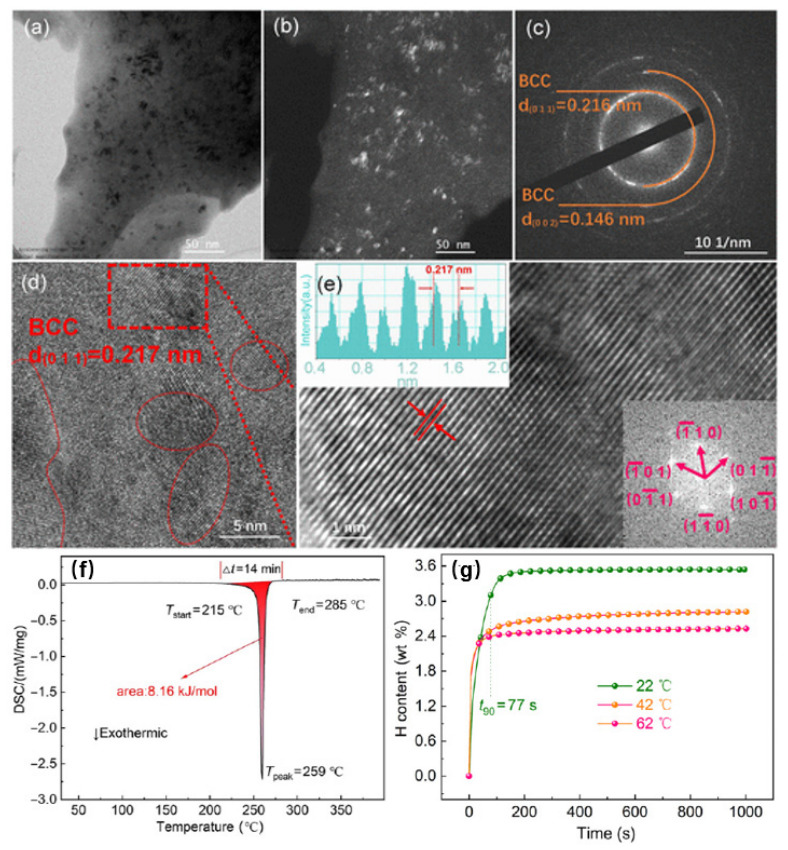
Representative mechanically processed HEA used here to illustrate how high-energy milling creates a defect-rich non-equilibrium hydrogen-hosting state with accelerated hydrogen uptake. (**a**–**e**) TEM, SAED, HRTEM, and FFT evidence of nanocrystalline microstructure in V_35_Ti_35_Cr_10_Fe_10_M_10_; (**f**) in situ hydriding DSC curve showing hydrogenation onset during heating with a distinct exothermic response below 300 °C; (**g**) hydrogen-absorption kinetic curves showing rapid uptake once the nanocrystalline state is established. Adapted from ref. [10].

If arc melting offers access to bulk hydride-forming HEAs, mechanical alloying and reactive ball milling provide a fundamentally different structural route. These methods are intrinsically non-equilibrium in nature. Rather than relying on high-temperature melting and solidification, they build alloys through repeated fracture, cold welding, severe plastic deformation, and intimate atomic mixing under continuous mechanical impact. The resulting materials are therefore often characterized by refined grain size, high defect density, large interfacial area, local strain accumulation, and, in some cases, partial amorphization or metastable phase formation. In this context, partial amorphization should not be treated as automatically beneficial or automatically detrimental. A limited loss of long-range order can, in some cases, broaden the distribution of accessible pathways and suppress abrupt bulk-like transformations, but excessive amorphization usually undermines pathway coherence by reducing crystallographic definition and making reversible hydrogen transport more difficult to sustain. It is therefore better understood as a route-dependent structural consequence whose value depends on whether it preserves or disrupts the continuity of the hydrogen-hosting state. For hydrogen storage, these features are highly consequential because they alter activation barriers, shorten diffusion distances, increase interface density, and may change both sorption kinetics and phase-transformation behavior [3,4]. The structural consequences of milling-based processing are illustrated by the representative V_35_Ti_35_Cr_10_Fe_10_M_10_ case in Figure 4, which is cited here to show how route-generated nanocrystallinity, interface density, and disorder translate into hydrogen-uptake behavior. High-energy processing can generate nanocrystalline HEAs with refined grains, abundant interfaces, and substantial local structural disorder, as evidenced by TEM, SAED, HRTEM, and FFT observations. These features are not merely descriptive microstructural details. They define the hydrogen-hosting state itself. Once such a fine-scale non-equilibrium structure is established, the alloy presents a dense network of grain boundaries and short diffusion distances that can facilitate hydrogen transport. This is consistent with the in situ hydriding DSC response shown in Figure 4f, where hydrogenation begins during heating and produces a clear exothermic event below 300 °C, indicating that the nanocrystalline alloy is more susceptible to hydrogen uptake than conventional coarse-grained BCC systems. The kinetic curves in Figure 4g further show that hydrogen absorption can proceed rapidly once this structural state has been established. The improved kinetics should not be attributed to increased surface area alone. Surface creation assists activation, but the more defensible mechanistic picture is that milling simultaneously shortens diffusion distances, increases internal defect density, and generates grain boundaries and interfaces that can serve as preferential ingress and short-range transport pathways. In the same sense, so-called defect-assisted storage should be understood primarily as a kinetic effect: defects and local strain fields may locally trap hydrogen and help the alloy approach equilibrium more rapidly, but they do not replace the dominant contribution of the bulk hydrogen-hosting phase to total storage capacity. The exothermic signal below 300 °C is therefore most reasonably interpreted as hydrogenation-driven hydride formation during heating, although in heavily milled materials a smaller overlap with recovery of stored strain cannot be excluded a priori. What remains critical is microstructural reversibility, namely whether this defect-rich nanocrystalline state persists in a functionally useful form during cycling or relaxes toward a less favorable host. For the present review, the importance of this example lies not simply in the fact that one alloy performs well, but in the more general lesson it conveys: mechanical processing does not merely produce powders or reduce particle size. It creates a distinct non-equilibrium microstructural regime in which nanocrystallinity, interface density, and local disorder become integral parts of hydrogen-storage function [10].

### 3.3. Other Preparation Routes and the Need for a Processing-Aware Perspective

Although arc melting and milling-based processing are the two most instructive routes for the present discussion, they do not exhaust the preparation landscape of hydrogen-storage HEAs. Additive manufacturing, electro-deoxidation-derived processing, sputtering-based deposition, and related routes are beginning to broaden the structural possibilities of these alloys by introducing new thermal histories, new spatial scales, and new non-equilibrium pathways. Their present value lies less in the number of hydrogen-storage examples than in the lesson they reinforce: in HEA hydrogen-storage research, preparation route is not merely a technical detail but a structural variable. The same nominal composition may end up as a chemically homogeneous bulk solid solution, a defect-rich nanostructured material, a metastable multiphase system, or some intermediate state that cannot be described adequately by composition alone. This is why any discussion of hydrogen-storage behavior that does not specify how the alloy was made remains incomplete at the level of mechanism. It is also why the next section must turn from preparation pathways themselves to representative alloy systems, where the consequences of preparation-dependent microstructures become easier to compare directly. Once route is recognized as a generator of structural state rather than only as a synthetic method, the comparison of hydrogen-storage HEAs can begin to move from alloy labels toward genuinely structural categories.

## 4. Representative High-Entropy Alloy Systems and Their Microstructure–Hydrogen-Storage Relationships

The influence of microstructure becomes most convincing when representative alloy systems are examined comparatively rather than in isolation. In the current literature, HEAs used in hydrogen-storage research do not constitute a single structurally uniform class. Some are bulk refractory alloys that act as hydride-forming hosts in their own right, whereas others are generated by strongly non-equilibrium processing and therefore exhibit fine-scale, defect-rich, or metastable structural states. A smaller number are introduced into Mg-based systems as modifiers rather than as the principal hydrogen-storage phase. What connects these otherwise different cases is that hydrogen-storage behavior cannot be read directly from nominal composition alone. Similar alloy families may show distinctly different activation behavior, hydrogen capacities, sorption kinetics, and reversible-storage responses once the structurally realized state changes. Representative systems are therefore useful here not as a catalog of materials, but as structurally resolved examples showing how preparation-dependent microstructures become measurable hydrogen-storage behavior.

### 4.1. Refractory HEAs as Hydride-Forming Materials

Refractory HEAs remain the clearest starting point for discussing microstructure–hydrogen-storage relationships because they often begin from comparatively simple bulk structural states while still displaying markedly different hydriding behavior. Systems such as TiZrNbHfTa and TiVZrNbHf are especially instructive in this regard. Both are obtained by arc-melting-based routes and both are commonly described as BCC-type starting alloys before hydrogenation, yet their hydrogen-storage behavior is not the same. TiZrNbHfTa shows a multistep absorption sequence involving formation of a monohydride region followed by further hydrogen-induced transformation, with a maximum capacity around 1.6 wt.% H_2_ under the reported conditions [11]. TiVZrNbHf, by contrast, shows a simpler BCC-to-BCT hydride pathway and reaches a higher maximum capacity of about 2.7 wt.% H_2_ [9]. Part of this contrast is chemical rather than purely microstructural. Replacing Ta with V changes not only hydrogen affinity but also the local distortion pattern and the electronic character of the BCC host, thereby altering which hydrogenated phase remains accessible and how readily the transformation pathway can remain coherent. This is why the present comparison should not be read as a strict route-controlled experiment. Rather, it shows that in refractory HEAs composition and microstructure must be interpreted together: Ta-containing systems may retain broader stability windows in the parent lattice but can also display stronger tendencies toward decomposition or pathway fragmentation at elevated temperature, whereas V-containing hosts more readily support direct hydride formation under the reported conditions. The relevant issue is therefore not simply which element is “better,” but how elemental substitution changes both the local lattice atmosphere and the hydrogen-induced transformation sequence. This comparison should not be read as a strict route-controlled experiment, because both alloys are arc-melted but not chemically identical. In particular, substitution of V for Ta changes hydrogen affinity and the stability window of the hydrogenated state, so the large capacity difference cannot be assigned to microstructure alone. The narrower point made here is that even within closely related arc-melted refractory HEAs, chemistry and structurally realized state must be interpreted together: elemental substitution shifts the thermodynamic landscape, whereas solidification history, retained strain, and local chemical bias determine how that landscape is actually traversed during hydrogenation and dehydrogenation. These differences are significant not simply because one alloy stores more hydrogen than another, but because they show that even within the same broad family of bulk refractory HEAs, hydrogen responds to the specific structural pathway available in the alloy rather than to a shared compositional label or a generic BCC description. This point becomes even clearer when refractory HEAs are considered more broadly. Their hydrogen-storage behavior is shaped not only by average phase constitution, but also by the local lattice environment, the degree of chemical mixing retained after solidification, and the stability of hydrogen-induced intermediate states. A relatively open BCC framework may favor hydrogen accommodation in general, but the actual absorption pathway, hydride stability, and reversibility still depend on how that framework is structurally realized. Refractory HEAs are therefore valuable in the present review not merely because they are among the best-known hydrogen-storage HEAs, but because they reveal under comparatively clean bulk conditions that composition does not act directly on storage behavior; it acts through microstructure. Similar route-sensitive behavior has been reported across other BCC-based refractory HEAs discussed in refs. [3,4], reinforcing that this is not an isolated comparison between two alloys but a broader family-level pattern.

### 4.2. Non-Equilibrium HEAs as Route-Defined Hydrogen Hosts

If refractory bulk alloys show that hydrogen-storage behavior already depends on structurally specific host states, non-equilibrium HEAs make the same point under a different structural regime. In these materials, the decisive features are no longer limited to average phase constitution in the conventional crystallographic sense, but include refined grain size, large interfacial area, local strain accumulation, high defect density, and the persistence of metastable structural states. Such characteristics directly affect hydrogen activation, short-range transport, and the pathways available during hydrogen absorption and desorption. As a result, mechanically processed HEAs cannot be regarded simply as powder forms of their bulk counterparts. They are structurally distinct hydrogen-hosting materials whose storage behavior reflects the state generated during processing. Strict paired comparisons between bulk and nanocrystalline states of the same nominal HEA remain relatively scarce in the current hydrogen-storage literature, and this limits how cleanly chemistry can be separated from route-generated structure. Even so, the available nanocrystalline examples remain instructive because they show which observables—especially activation and uptake kinetics—respond first when grain scale, defect density, and interface population are strongly redistributed by mechanical processing. The nanocrystalline V_35_Ti_35_Cr_10_Fe_10_M_10_ system summarized in Figure 4 is instructive in this respect. High-energy processing produces a fine-scale BCC-based alloy with abundant interfaces and short diffusion distances, while TEM, SAED, HRTEM, and FFT observations show that the resulting microstructure is not a trivial refinement of a bulk host, but a distinct nanocrystalline structural state [10]. The in situ hydriding DSC response further indicates that hydrogenation begins during heating with a clear exothermic event below 300 °C, and the kinetic curves show that hydrogen uptake can proceed rapidly once this structural state is established [10]. The important point here is not only that one alloy exhibits favorable kinetics. It is that the structural identity of the material itself has become route dependent. What distinguishes the nanocrystalline state mechanistically is not merely the reduction in particle size. Grain refinement, retained strain, and interface multiplication together redistribute the hydrogen-hosting environment by increasing the density of energetically non-equivalent local regions. As a result, the kinetic gain cannot be reduced to faster surface access alone; it reflects a coupled change in transport distance, barrier distribution, and pathway availability within the non-equilibrium host. Once non-equilibrium processing is introduced, the operative hydrogen-hosting environment is defined by nanocrystallinity, interface density, and retained local disorder rather than by nominal formula alone. Once non-equilibrium processing is introduced, the operative hydrogen-hosting environment is defined by nanocrystallinity, interface density, and retained local disorder—here referring primarily to local atomic positional disorder and strain fields, together with incomplete short-range chemical equilibration—rather than by nominal formula alone. Related observations across other milling-derived HEA systems discussed in refs. [3,4] support the same conclusion: once synthesis generates nanocrystallinity, high defect density, and abundant interfaces, hydrogen-storage behavior becomes inseparable from the retained non-equilibrium state. For this reason, non-equilibrium HEAs are especially important to a microstructure-centered review. They make it impossible to sustain the idea that composition alone is an adequate descriptor of hydrogen-storage behavior. In such systems, hydrogen does not respond to an alloy formula in the abstract, but to a route-defined environment composed of specific phase assemblages, interface densities, strain states, and defect populations. This is also why statements such as “milling improves kinetics” remain structurally incomplete unless they are tied to the actual microstructural changes generated by the route.

### 4.3. HEAs as Modifiers in Mg-Based Systems

A smaller but still highly relevant branch of the literature concerns the use of HEAs as modifiers in Mg-based hydrogen-storage systems. This direction should not be taken as the main identity of the present review, because here the HEA is no longer the dominant hydrogen-storage phase. Even so, it remains a useful extension of the same structural logic. Once an HEA is introduced into MgH_2_-based materials, its role depends less on nominal composition alone than on the structurally realized state that it brings to the composite, including its phase constitution, particle size, defect density, chemical homogeneity, and, above all, the nature of the HEA/MgH_2_ interface. One practical reason HEAs are attractive as modifiers is not simply configurational complexity in the abstract, but the possibility of combining multicomponent catalytic sites with interface-rich morphologies that remain functionally active in the 300–350 °C window relevant to MgH_2_. That said, the present literature should not yet be read as proving that HEA modifiers universally preserve nanostructure better than all conventional catalysts under these conditions. The more defensible conclusion is that their multicomponent chemistry and structurally robust interface states can make them especially competitive when catalytic transfer and cycling persistence must be balanced. Recent studies on FeCoNiCrTi-, CrCoNi-, and FeCoNiCrMo-based systems make this point clear. In FeCoNiCrTi-promoted MgH_2_, the catalytic effect is commonly discussed in terms of a hydrogen-pumping mechanism, yet the actual benefit still depends on the structurally realized alloy state and on how that state mediates hydrogen transfer across the interface [12]. In CrCoNi-based nanosheet additives, the observed enhancement of MgH_2_ dehydrogenation and rehydrogenation cannot be reduced to the statement that “a medium-entropy alloy works as a catalyst”; it depends on the fine-scale morphology and the interface-rich state produced during preparation [13]. A similar conclusion emerges from FeCoNiCrMo nanosheet systems, where the catalytic improvement is inseparable from the route-generated high-surface-area sheet-like structure and the resulting contact state with MgH_2_ [14]. In that sense, this class of systems does not redirect the present review toward Mg-based catalysis as a separate topic. It simply reinforces the same central conclusion reached from hydride-forming HEAs themselves, namely that hydrogen-storage behavior is governed by the structural state actually created during preparation. Comparable behavior reported for other HEA-assisted MgH_2_ systems suggests that the decisive variable is not the nominal HEA formula alone, but the interfacial state created during synthesis, including nanosheet exposure, defect-rich surface regions, and contact geometry with MgH_2_.

Taken together, the representative systems discussed above support three transferable microstructure–hydrogen-storage relationships. In arc-melted bulk HEAs, the decisive link lies in phase constitution and hydrogen-induced lattice transformation, because these variables govern hydride pathway selection and reversible storage behavior. In mechanically processed HEAs, the dominant link shifts to nanocrystallinity, defect population, and interface density, since activation and short-range hydrogen transport are controlled more directly by the retained non-equilibrium structural state. In HEA-modified Mg-based systems, the key relationship is primarily interfacial rather than bulk-hydride thermodynamic, with surface accessibility, defect-rich contact regions, and catalytic hydrogen exchange governing the observed kinetic response. Across all three groups, composition defines the available chemical space, but the operative hydrogen-hosting or hydrogen-mediating state is determined by the structural condition actually created during preparation. Meaningful comparison across the field therefore requires moving from alloy labels toward structurally explicit descriptions, which in turn leads directly to the next section, where the focus shifts to the descriptors that recur across these systems and to the unresolved problem of why their use remains inconsistent from one study to another.

## 5. Key Microstructural Descriptors and Unresolved Correlations

If the preceding sections establish that preparation route determines microstructure and that microstructure, in turn, governs hydrogen-storage behavior, the next question is which structural descriptors are actually useful for comparing different HEA systems. This is precisely where the field remains unsettled. A large number of studies already report hydrogen capacity, activation behavior, plateau pressure, sorption kinetics, or reversibility, yet the structural variables used to interpret those results are far less consistent. A descriptor-centered discussion becomes necessary at this stage because the field increasingly seeks transferable structure-property relationships rather than continued composition screening alone. Yet the current literature still applies structural descriptors unevenly. In some systems, discussion remains focused almost entirely on nominal composition; in others, phase constitution is emphasized while grain size, defect density, interface density, or local chemical heterogeneity are invoked only selectively. As a result, structurally similar alloys are not always compared on the same basis, and alloys with comparable hydrogen-storage responses are often explained through different vocabularies. Table 1 is introduced here as a comparative device rather than as a list of abstract HEA design parameters. For this reason, the discussion below places greater emphasis on descriptors that can be compared quantitatively across systems, such as grain scale, phase constitution, hydrogen-storage capacity, and representative kinetic or thermal-response metrics, rather than relying only on qualitative route labels. To make the route–microstructure link more explicit, the table below includes not only structurally realized state and representative storage response, but also the morphological or grain-scale information reported in the corresponding literature where available. It aligns representative systems with preparation route, structurally realized state, hydrogen-storage response, and the descriptor levels most relevant to interpretation. In that form, the table serves the central argument of this perspective more directly, because it links the representative cases discussed above to the descriptor-centered analysis that follows.

### 5.1. Phase Constitution and Metastability

Among the available descriptors, phase constitution remains the most fundamental because it defines the broad type of hydrogen-hosting environment presented by the alloy. Whether an HEA exists as a single BCC solid solution, a multiphase system, an intermetallic-containing alloy, or a metastable non-equilibrium structure directly affects hydrogen accommodation, hydride-formation pathway, and reversibility [3,4,7,8]. Yet phase constitution alone is not enough. Two alloys may both be described as BCC-based and still behave very differently because the effective hydrogen-hosting environment depends on more than average symmetry. Local lattice distortion, chemical disorder, minor-phase content, and the stability of hydrogen-induced intermediate states all influence hydrogen occupancy and the structural response of the host lattice during absorption. This is why descriptor systems based only on average phase labels tend to lose explanatory power as the literature becomes more diverse. A useful structural description must distinguish not only what phase is present, but what kind of hydrogen-hosting environment that phase actually provides.

Metastability makes this issue even more important. Many of the most relevant HEA systems in hydrogen-storage research are not ideal equilibrium materials, but products of arc melting followed by homogenization, mechanical alloying, or reactive ball milling, each of which leaves a distinct structural signature. In such systems, the meaningful descriptor is not simply whether the alloy is nominally BCC, FCC, intermetallic, or amorphous, but whether that state is stable, metastable, or already evolving under hydrogen exposure. Phase constitution is therefore indispensable, but only when read together with structural stability and transformation tendency.

### 5.2. Local Lattice Distortion and Hydrogen Occupancy

If phase constitution defines the broad hydrogen-hosting framework, local lattice distortion and hydrogen occupancy determine how that framework is chemically experienced by hydrogen. In a multicomponent HEA, hydrogen does not encounter a single ideal interstitial site, because atomic-size mismatch, local chemical disorder, and differences in bonding preference generate a distribution of site geometries and site energies. Some local environments expand interstitial volume or soften the lattice and therefore lower the cost of hydrogen insertion, whereas others destabilize occupancy by imposing steric or electronic penalties. The practical consequence is that hydrogen uptake in HEAs is governed not only by average crystal symmetry but also by the statistical landscape of local environments available within that symmetry. In this sense, parameters such as atomic-size difference, electronegativity difference, mixing enthalpy, and valence electron concentration are useful only insofar as they help explain why particular local occupancy states become favorable and why hydrogen-induced structural response differs from one alloy to another.

**Figure 5:**
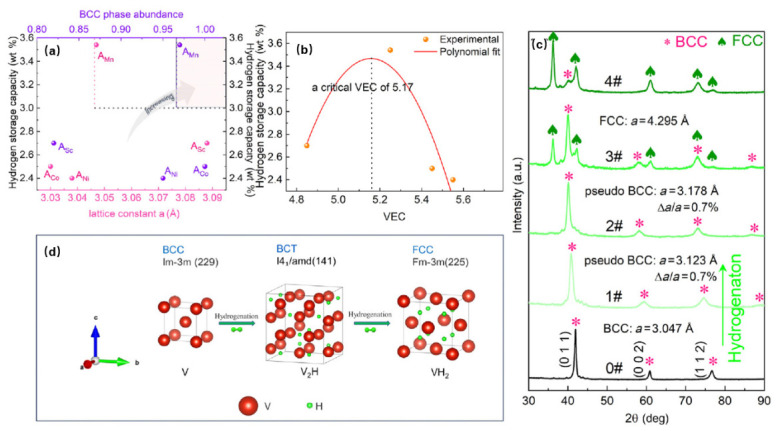
Representative descriptor-level example used here to show how local structure, hydrogen occupancy, and hydrogen-induced transformation are coupled in an HEA system. (**a**) Dependence of hydrogen-storage capacity on the abundance and lattice constant of the main hydrogen-absorbing phase; (**b**) hydrogen-storage capacity as a function of valence electron concentration; (**c**) XRD evidence of phase evolution with increasing hydrogen uptake; (**d**) schematic structural evolution during hydrogenation. Adapted from ref. [10].

This is also why chemistry must remain visible within a microstructure-centered framework. Local bonding heterogeneity affects hydrogen binding strength, local strain fields influence interstitial accessibility, and the coupling between occupancy and lattice response determines whether absorption proceeds mainly by progressive site filling or by a more collective structural transformation. As shown in Figure 5, variables such as hydrogen-absorbing-phase abundance, lattice constant, valence electron concentration, and phase-evolution pathway do not operate independently. Together they define the effective occupancy landscape and the structural response that accompanies hydrogen uptake. Descriptor-level interpretation in HEA hydrogen storage therefore cannot stop at isolated quantities such as phase label or lattice distortion alone; it must track how local chemistry, local geometry, and the transformation pathway converge within an evolving hydrogen-hosting state.

### 5.3. Grain Size, Defect Density, and Interface Density

A second group of descriptors concerns the fine structure of the alloy, especially grain size, defect density, and interface density. These variables become particularly important in mechanically alloyed and reactively ball-milled HEAs, where hydrogen-storage behavior is often governed as much by the non-equilibrium structural state as by the nominal phase constitution [9,10,11,12,13,14]. Grain refinement shortens diffusion distances, defect-rich regions can provide kinetically favorable pathways for hydrogen ingress and egress, and increased interface density may alter both transport and phase-transformation behavior. In this sense, these descriptors operate at a structural scale below average phase constitution but above atomistic site occupancy: they describe the mesoscopic architecture through which hydrogen moves. The difficulty is that these variables are often invoked unevenly and sometimes interchangeably. One study may attribute improved kinetics to nanocrystallinity, another to lattice strain, another to severe deformation, and another simply to the route of ball milling itself. All of these factors may be relevant, but they do not describe the same thing. Nanocrystallinity refers primarily to a length-scale condition; defect density refers to the population of non-ideal structural features; interface density concerns the abundance of boundaries or phase contacts; and route name is not, by itself, a descriptor at all. For the present review, the most useful way to treat these variables is hierarchically. Grain size describes the scale of the host architecture, defects describe the density of local non-equilibrium features embedded within it, and interface density describes the number of structural junctions through which hydrogen must move or across which transformation must proceed. Once these distinctions are made, comparisons across different HEA systems become less ambiguous.

### 5.4. Chemical Homogeneity and Local Partitioning

A further descriptor set concerns the distribution of chemical species within the alloy: whether the material is chemically homogeneous at the structurally relevant scale or whether it contains local partitioning, segregation, or hidden compositional fluctuation. This issue is especially important because many HEAs are still described using broad labels such as “single-phase” or “solid solution,” which can easily obscure chemically distinct local environments. From a hydrogen-storage perspective, however, hydrogen does not encounter an average composition. It encounters specific local neighborhoods that determine interstitial-site energy, local lattice stiffness, hydrogen affinity, and the likelihood of hydrogen-induced structural change. Even when diffraction suggests a simple average phase constitution, local partitioning may still alter site hierarchy, transformation sequence, and reversibility. Chemical homogeneity should therefore not be treated merely as a compositional ideal, but as a microstructural descriptor with direct hydrogen-storage implications. A practical boundary may be drawn as follows: intrinsic chemical disorder refers to statistically distributed local compositional variation retained within a structurally coherent host, whereas undesirable segregation refers to spatial partitioning into compositionally biased regions large enough to create discontinuous site hierarchies, transport barriers, or distinct phase behavior. This becomes even more important when bulk refractory systems are compared with strongly non-equilibrium materials. In arc-melted alloys, nominally homogeneous bulk phases may still retain local chemical bias inherited from solidification and post-treatment history, whereas in mechanically processed systems severe deformation and incomplete local equilibration may generate even more pronounced heterogeneity. The result is that two alloys sharing the same nominal formula and even the same broad phase label may still present hydrogen with different chemically resolved local environments. A more transferable framework will require greater precision here, because without that precision microstructure will continue to be described too coarsely for robust structure–property comparison.

### 5.5. Processing-History Dependence and Descriptor Inconsistency

The preceding descriptors all point toward a final and overarching one: processing-history dependence. In practice, this is not a separate descriptor at the same level as phase constitution or defect density, but the structural condition that makes all of them route sensitive. Preparation route determines which phases appear, how much strain is retained, how interfaces are generated, whether metastable states persist, and how chemically homogeneous the final alloy actually becomes. For that reason, although the early HEA concept emphasized multicomponent alloy design and the formation of compositionally complex solid solutions [15], hydrogen-storage HEAs should not be compared as if nominal composition alone defined the material. The structurally realized state always carries the imprint of processing history [3,4,9,10,11,12,13,14]. This is particularly important because many of the most informative HEA hydrogen-storage systems are route dependent by construction. Arc melting, post-homogenization, mechanical alloying, and reactive ball milling do not simply produce the same alloy in different physical forms; they create different structural states with different hydrogen-hosting or hydrogen-mediating environments. Seen in this way, the main unresolved difficulty in the field is not the absence of candidate descriptors, but the lack of comparability among them. Structural parameters such as atomic-size mismatch, mixing enthalpy, or related phase-selection metrics remain useful as broad guides [16,17,18,19,20,21], but they do not, by themselves, specify the actual microstructure that a particular preparation pathway will produce, nor do they uniquely determine hydrogen-storage response [5,6,7,8]. What the field still lacks is a transferable framework that can connect preparation, microstructure, and hydrogen-storage behavior across different alloy families in a way that is not entirely system specific.

In practical terms, this means that future work must move beyond asking which composition performs best and toward asking which structural descriptors remain meaningful across different preparation routes and different classes of HEAs. Until that question is addressed more systematically, the literature will continue to generate valuable individual case studies, but genuine structure–property generalization will remain limited. That unresolved gap leads directly to the final section, because it also defines the main challenge for the future development of HEAs in hydrogen storage. Taken one step further, the descriptor problem is not only which variables matter, but which variables remain dominant in which structural regime. In bulk refractory hydride-forming HEAs, phase constitution, metastability, and hydrogen-induced transformation sequence are the most informative descriptors. In mechanically processed non-equilibrium HEAs, grain size, defect density, retained strain, and interface population become more decisive because they govern activation and short-range hydrogen transport. In HEA-modified MgH_2_ systems, the most meaningful descriptors are interfacial accessibility, catalytic contact state, and defect-rich surface chemistry. A transferable framework therefore cannot rely on one universal descriptor set applied uniformly to all HEA hydrogen-storage systems. It must match descriptor hierarchy to preparation-defined structural regime.

## 6. Conclusions and Outlook

This review has argued that the most meaningful way to understand hydrogen storage in high-entropy alloys is not through nominal composition alone, but through the microstructural states that different preparation pathways actually produce. In this sense, HEAs should not be regarded simply as a vast compositional library for hydrogen-storage screening. Their real scientific value lies in the fact that they provide access to a wide spectrum of structurally distinct hydrogen-hosting states, ranging from comparatively simple bulk refractory solid solutions to defect-rich, metastable, and interface-dominated non-equilibrium materials [22,23,24]. What hydrogen encounters in practice is not an abstract alloy formula, but a route-generated structural environment. Seen from this perspective, preparation route is not a synthetic afterthought. It is the upstream variable that determines which phases form, how local environments are organized, how much strain and disorder are retained, how interfaces are distributed, and how chemically homogeneous the final alloy actually becomes. Once that point is recognized, a more general conclusion follows: the hydrogen-storage behavior of HEAs cannot be interpreted adequately unless preparation, microstructure, and storage response are discussed as one connected hierarchy. Composition defines the available chemical space, but microstructure determines how that space is physically realized. This shift in viewpoint is especially important for comparing different classes of HEAs. Bulk refractory hydride-forming alloys, mechanically processed nanocrystalline systems, and HEA-modified Mg-based composites may appear to belong to the same broad materials family, yet they operate through very different structural states. What unifies them is not a shared phase label or a generic appeal to multicomponent synergy, but the fact that hydrogen-storage performance emerges from the structurally specific environment created during preparation. That is why route-dependent microstructure provides a more transferable framework for discussion than nominal composition alone.

At the same time, the field remains limited by the way structural descriptors are still used. Parameters such as phase constitution, local lattice distortion, hydrogen occupancy, grain size, defect density, interface density, and chemical homogeneity are all potentially informative, but they are not yet used in a sufficiently coherent or comparable way across the literature. In some studies, average phase labels still dominate interpretation; in others, local structural variables are invoked selectively or qualitatively, making it difficult to compare systems on a common structural basis. The central challenge is therefore no longer only to discover more promising HEA compositions, but to establish which descriptors remain meaningful across different preparation routes and different classes of hydrogen-storage alloys.

Future progress will depend on moving beyond composition-driven trial and error toward a more explicit microstructure–hydrogen-storage framework. This requires closer attention to how structural states are generated, how they evolve during hydrogen absorption and desorption, and how descriptor-level comparisons can be made across systems that are not prepared in the same way. A particularly important next step will be dynamic characterization, including in situ or operando diffraction, scattering, thermal analysis, and local-environment probes, because static pre-hydrogenation descriptions are often insufficient once hydrogen actively reshapes the host lattice. For new readers entering the field, a practical starting point is to evaluate any candidate HEA in the following order: first the realized phase constitution after preparation, then the degree of retained strain/disorder and chemical homogeneity, then the hydrogenation pathway and its reversibility under cycling, and only after that the headline capacity value. It also requires greater caution in assigning mechanisms. Statements such as “ball milling improves kinetics” or “BCC structure favors hydrogen storage” remain incomplete unless they are tied to the actual microstructural conditions that hydrogen experiences. More rigorous comparison will therefore depend on combining route-aware synthesis, structurally resolved characterization, and hydrogen-storage measurements that are interpreted at the same structural scale. In this sense, the main limitation of current HEA hydrogen-storage research is not the lack of promising materials, but the absence of a sufficiently unified language for describing why different materials behave differently. The most useful contribution of a microstructure-centered perspective is that it makes this limitation visible. Once the field begins to compare HEAs not only as compositions but as preparation-defined structural states, more transferable structure–property relationships should become possible. That shift is likely to be more important for long-term progress than continued expansion of composition space alone.

## Figures and Tables

**Table 1:** Representative high-entropy alloy systems discussed in this perspective, compared by preparation route, phase constitution/morphology, grain-scale structural state, representative hydrogen-storage metrics, and the microstructural implications they reveal.

Representative System	Preparation Route	Phase Constitution/Morphology	Grain Size/Structural Scale	Representative Hydrogen-Storage Metric	Microstructural Implication	Ref.
TiVZrNbHf	Arc melting	Single-phase BCC bulk alloy; hydrogenation leads to a BCT-type hydride state	Bulk cast host; coarse-grained/macroscopic ingot scale	Hydrogen absorption starts near 299 °C under low H_2_ pressure; maximum capacity reaches about 2.7 wt.% at elevated pressure	Bulk phase constitution and reversible lattice transformation dominate storage behavior	[9]
TiZrNbHfTa	Arc melting	BCC parent alloy with pressure-dependent transformation to BCT/FCC hydride-related states	Bulk cast host; chemically homogenized bulk state; grain-scale details not consistently quantified in the perspective sources	Maximum hydrogen-storage capacity is about 1.6–1.65 wt.% H_2_; storage behavior is strongly coupled to hydride pathway selection	Refractory chemistry and phase-stability window jointly define pathway integrity	[11]
V_35_Ti_35_Cr_10_Fe_10_M_10_ (M = Ni, Cu, Co)	Mechanical alloying	Nanocrystalline BCC-dominated state with high defect density and refined domains	Nanocrystalline/fine powder; grain-refined, interface-rich state	Markedly accelerated room-temperature hydrogen uptake after activation, highlighting strong route sensitivity of kinetics	Nanocrystallinity, defect population, and shortened diffusion length govern the kinetic response	[10]
FeCoNiCrTi + MgH_2_	Composite catalytic route	HEA-derived modifier generating multicomponent interfacial active sites in MgH_2_	Fine interfacial additive/composite contact state	Dehydrogenation temperature is reduced and sorption kinetics are improved relative to undoped MgH_2_	Interfacial catalysis and hydrogen-pumping effect	[12]
CrCoNi-based nanosheets + MgH_2_	Interface-engineered composite	Nanosheet modifier with large exposed surface and abundant contact interface	Two-dimensional nanosheet morphology/high surface accessibility	Hydrogen desorption and rehydrogenation of MgH_2_ are significantly accelerated by morphology-controlled interface mediation	Morphology-controlled interfacial transport and nucleation	[13]
FeCoNiCrMo nanosheets + MgH_2_	Interface-engineered composite	Defect-rich nanosheet catalyst with high surface accessibility	Sheet-like nanostructure/interface-rich state	MgH_2_ shows improved kinetic response and better cycling-related structural persistence	Interface density, defect chemistry, and catalytic-site accessibility	[14]

Note: Grain-size or morphology information is reported here at the level available in the corresponding literature emphasized in the perspective; where exact numerical values are not consistently provided in the cited source used in the manuscript, the structural scale is described qualitatively to preserve comparability across systems.

## Data Availability

No new data were created or analyzed in this study. Data sharing is not applicable.
